# Plasticity of repetitive sequences demonstrated by the complete mitochondrial genome of *Eucalyptus camaldulensis*

**DOI:** 10.3389/fpls.2024.1339594

**Published:** 2024-03-27

**Authors:** Yoshinori Fukasawa, Patrick Driguez, Salim Bougouffa, Karen Carty, Alexander Putra, Ming-Sin Cheung, Luca Ermini

**Affiliations:** ^1^ Center for Bioscience Research and Education, Utsunomiya University, Utsunomiya, Japan; ^2^ Core Labs, King Abdullah University of Science and Technology, Thuwal, Saudi Arabia; ^3^ Computational Bioscience Research Center, King Abdullah University of Science and Technology, Thuwal, Saudi Arabia; ^4^ NORLUX NeuroOncology Laboratory, Department of Cancer Research, Luxembourg Institute of Health, Luxembourg, Luxembourg

**Keywords:** mitochondrial genome, eucalyptus, repeat sequences, homologous recombination, nested repeat, isomer

## Abstract

The tree *Eucalyptus camaldulensis* is a ubiquitous member of the *Eucalyptus* genus, which includes several hundred species. Despite the extensive sequencing and assembly of nuclear genomes from various eucalypts, the genus has only one fully annotated and complete mitochondrial genome (mitogenome). Plant mitochondria are characterized by dynamic genomic rearrangements, facilitated by repeat content, a feature that has hindered the assembly of plant mitogenomes. This complexity is evident in the paucity of available mitogenomes. This study, to the best of our knowledge, presents the first *E. camaldulensis* mitogenome. Our findings suggest the presence of multiple isomeric forms of the *E. camaldulensis* mitogenome and provide novel insights into minor rearrangements triggered by nested repeat sequences. A comparative sequence analysis of the *E. camaldulensis* and *E. grandis* mitogenomes unveils evolutionary changes between the two genomes. A significant divergence is the evolution of a large repeat sequence, which may have contributed to the differences observed between the two genomes. The largest repeat sequences in the *E. camaldulensis* mitogenome align well with significant yet unexplained structural variations in the *E. grandis* mitogenome, highlighting the adaptability of repeat sequences in plant mitogenomes.

## 1 Introduction

Eucalyptus trees, known for their rapid growth rates, are increasingly recognized for their significant contribution to a sustainable economy. This is marked by the numerous nuclear reference genome assemblies of the *Eucalyptus* genus registered in NCBI (Myburg et al., 2014; Wang et al., 2020; Driguez et al., 2021; Lötter et al., 2022). Among several hundred species within this genus, *Eucalyptus camaldulensis*, river red gum, has the broadest geographical distribution (Butcher et al., 2002).

Using PacBio high-accuracy long-read (HiFi) sequencing, we have previously assembled the nuclear genome of *E. camaldulensis* and demonstrated that chromosome-scale assembly is feasible (Driguez et al., 2021). In contrast to the continuous assembly of the nuclear genome, there were numerous fragmented sequences that resembled organelle genomes. Due to the high degree of fragmentation, the mitogenome has not yet been determined. This issue extends beyond *E. camaldulensis* and represents a common challenge across different species of eucalypts. While multiple nuclear genomes are available, only one fully annotated mitogenome for *E. grandis* exists within the *Eucalyptus* genus (Pinard et al., 2019).

The mitochondria provide numerous essential pathways for eukaryotic cells, despite the fact that the majority of proteins are encoded by nuclear genomes (Fukasawa et al., 2015), which reflects the smaller size of mitochondrial genomes. Plant mitogenomes exhibit diverse evolutionary patterns, complicating assembly efforts despite their small size. Angiosperm mitogenomes show a considerable variation in size and gene content, ranging from 66 kb to 11.3 Mb, in contrast to the relatively stable size of plastid genomes (Sloan, 2013; Sun et al., 2022). Although angiosperm mitogenomes have low sequence evolution rates, the synteny conservation rates remain low due to frequent genome rearrangements (Cole et al., 2018). Such rearrangements, resulting from homologous recombination, play a significant role in plant mitogenome evolution.

Cross-ecotype comparisons of *Arabidopsis* plants revealed that disrupting the plant-specific *MSH1* gene results in stoichiometric changes and that polymorphisms among *Arabidopsis* ecotypes are caused by *MSH1*-regulated recombination (Arrieta-Montiel et al., 2009). A recent report also emphasizes the crucial role of *MSH1* in mitigating mutation rates in plant organelles (Wu et al., 2020). This distinctive genetic feature in plants indicates the high occurrences of homologous recombination-based rearrangements in plant mitogenomes, highlighting the need for analytical approaches different from those used for nuclear genomes (Fischer et al., 2022). The intricate rearrangement patterns observed in the study suggest the need for further methodological innovations in order to fully understand the dynamic nature of plant mitogenomes.

In this study, we assembled, for the first time, the complete mitogenome of *E. camaldulensis* using PacBio HiFi sequencing. Further validation of the assembled sequence was conducted using Oxford Nanopore Technologies (ONT) sequencing, which confirmed the existence of multiple forms of the mitogenome. Following the chloroplast/plastid (GenBank: CM024681.1) and nuclear (Driguez et al., 2021) genomes, the mitogenome of *E. camaldulensi*s, as presented in this study, represents the final piece of the entire genetic information of the species.

## 2 Materials and methods

### 2.1 Genome assembly and annotation of mitogenome

PacBio Sequel-II raw subread data generated from the whole genome of *E. camaldulensis* was downloaded from NCBI and was used for the mitogenome assembly (SRX11929917). High Fidelity Circular Consensus reads (HiFi CCS) were locally called from the downloaded subread data using SMRTLink ver. 11.1. The first *de novo* assembly was computed using hifiasm ver. 0.16.1 (Cheng et al., 2021) against the whole PacBio HiFi dataset. Hereafter we refer to contigs computed by hifiasm as those computed with the whole-genome sequencing dataset, including nuclear genome assembly (Driguez et al., 2021). We searched mitogenome contigs with homology to the mitogenome of *E. grandis* (NCBI reference sequence: NC_040010.1). Because the collected contigs show different forms of the sequences, rearrangements mediated by repeat sequences were considered to explain multiple contigs (Sloan, 2013). Therefore, a repeat-aware graph assembler was applied to suppress this issue (Fischer et al., 2022). To accelerate computational time, mitochondria-derived reads were extracted from the original dataset using the *E. grandis* genome with minimum query coverage to the reference at 70% using minimap2 ver.2.26 (Li, 2018). We assembled the *E. camaldulensis* mitogenome using Flye ver. 2.8.3 with default parameters (Kolmogorov et al., 2019). The assembled mitogenome contig was annotated by using MITOFY ver. March 22, 2012 (Alverson et al., 2010) and tRNAscan-SE ver. 2 (Chan et al., 2021) with manual corrections to confirm the annotation. The genome circle map was drawn by using ORGDRAW ver. 1.3.1 (Greiner et al., 2019). The mitochondrial reads were reextracted from the entire dataset by mapping against reference sequences containing our mitogenome assembly, the nuclear genome (GCA_019915185.1), and the plastid genome (CM024681.1) for further analysis.

### 2.2 Repeat analysis

The direct repeat (DR) and inverted repeat (IR) contents of the *E. camaldulensis* mitogenome were analyzed using ROUSfinder ver. 2.0 and blastn ver. 2.14.1 (Altschul et al., 1997; Wynn and Christensen, 2019). Validation of the repeat-mediated genomic rearrangement was performed by mapping the mitochondria-derived reads against all possible sequences with the target repeat in the middle. Specifically, for each repeat pair detected by ROUSfinder, 3,000-bp flanking sequences upstream and downstream of the repeat sequence were extracted, and four sequences representing possible isomers mediated by homologous recombination were generated with the extracted flanking and the target repeat sequences. These generated sequences were combined as a reference set. The mitochondria-derived reads were mapped to the reference sequence set using minimap2 ver. 2.26, and supporting reads were counted for each repeat pair if a read is mapped to a sequence with at least 1,800-bp overlap to flanking sequences in both ends as a primary alignment. A few repeats are nested in another larger repeat sequence; however, the 1,800-bp threshold is long enough to detect unique sequences beyond the repeat sequence covering the target nested repeat (the largest covering repeat sequence has 1,467 bp in length). The assembly used for the repeat analysis was named Master Circle 1 form (MC1). It should be noted that because the rates of rearrangement mediated by the long (>1,000 bp) repeat pairs were significant, we designated the rearranged MC1 sequence mediated by the longest repeat (repeat-1) as Master Circle 2 form (MC2). Additionally, the sequences of MC1 and MC2 rearranged by the second longest repeat (repeat-2) were termed MC1^2^ and MC2^2^, respectively.

### 2.3 Sequence comparison

The mitogenomes of *E. grandis* and *E. camaldulensis* were compared using LAST ver. 1454 (Kiełbasa et al., 2011) with default parameters. The MC2 of the *E. camaldulensis* mitogenome was selected as a representative sequence due to the least number of variations compared to the *E. grandis* mitogenome. Detected significant hits were visualized and summarized using the maf-convert command. Large rearrangements were visualized by using SyRI (Goel et al., 2019). An E-value of 1e-20 was used as the threshold.

### 2.4 Phylogenetic analysis

The taxonomic placement of *E. camaldulensis* was established by analyzing the complete mitochondrial genomes of all plant species within the *Myrtaceae* family available in the NCBI database. The complete genomes of *E. grandis*, *Syzygium samarangense*, and *Rhodomyrtus tomentosa* (accession numbers: NC_040010.1, NC_079700.1, and NC_071968.1, respectively) were downloaded. The complete mitochondrial genome of *Begonia coptidifolia* (accession number: LC706752.1) was used as an outgroup for rooting purposes. A multiple sequence alignment of five mitochondrial genomes, including *E. camaldulensis*, was generated using progressiveMauve with default parameters (Darling et al., 2010). Phylogenetic relationships were reconstructed using maximum likelihood with the general time reversible model. Initial trees for the heuristic search were obtained automatically by applying neighbor-joining and BioNJ algorithms to a matrix of pairwise distances estimated using the maximum composite likelihood approach and then selecting the topology with a superior log likelihood value. To model differences in the rates of evolution between sites, a discrete Gamma distribution was used. Positions containing missing/ambiguous data were excluded. An estimate of support for each branch was determined with the bootstrap test using 100 pseudoreplications. Phylogenetic analyses were conducted in MEGA11 (Tamura et al., 2021).

### 2.5 Sample collection and HMW DNA extraction

Fresh *E. camaldulensis* leaves were collected from the research greenhouse at King Abdullah University of Science and Technology, Thuwal, Saudi Arabia. HMW DNA was extracted by following a modified Qiagen protocol (dx.doi.org/10.17504/protocols.io.bafmibk6).

### 2.6 Oxford nanopore sequencing

This study utilized the GridION sequencer for Oxford Nanopore sequencing of *E. camaldulensis*. In preparation for library creation, short fragments were removed from the extracted HMW DNA using either BluePippin with a 30-kb cutoff or the Short Read Eliminator XL kit (SRE XL, Circulomics, Baltimore, MD, USA) as per the manufacturer’s instructions. The size-selected DNA underwent a cleanup process with AMPure XP beads, followed by library processing using the genomic DNA by ligation protocol (SQK-LSK109, Oxford Nanopore, Oxford, UK). Specifically, DNA samples of *E. camaldulensis* (1.3 μg) were repaired and end-prepped before bead cleanup and adapter ligation. The ligated product underwent bead cleaning with long fragment buffer (LFB) and was subsequently eluted. The prepared library for *E. camaldulensis* (8 fmol) was loaded onto the GridION platform (FLO-MIN106D). This study extracted mitochondrial reads from the ONT dataset using the same criterion applied for the HiFi dataset. The duplex-like reads (i.e., reads mappable to the same region on the reference in two strands) were removed because they cannot be mapped lineally.

### 2.7 Validation of repeat-mediated rearrangements

The mitochondrial reads in both HiFi and ONT datasets were mapped against MC1 sequence. Only the reads that had 95% of their bases aligned linearly with the MC1 sequence were retained for coverage computation. Reads that were partially mapped, i.e., those with less than 95% of their bases were aligned linearly, were mapped against an alternative sequence, MC2^2^. This was done if a portion of the reads fully and linearly aligned with the alternative form but not with the reference sequence. To filter reads, CoverM ver. 0.7.0 was utilized (https://github.com/wwood/CoverM).

## 3 Results

### 3.1 Structure of *E. camaldulensis* mitochondria genome

**Figure 1:**
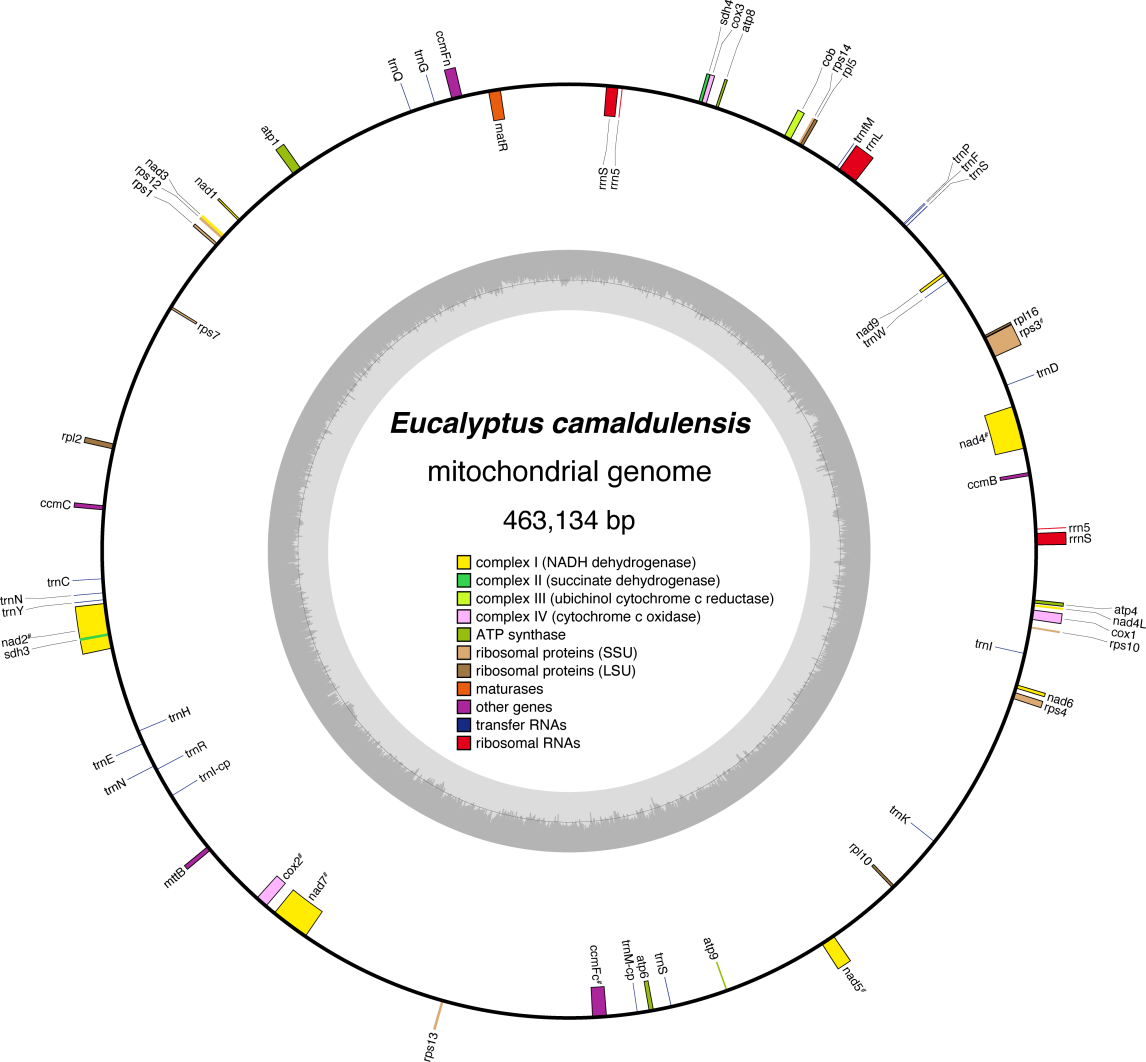
Circular map of the *E. camaldulensis* mitochondrial genome. Genes inside the circle represent genes transcribed clockwise, and genes outside the circle are transcribed counterclockwise. Genes are categorized based on their functional classification, and the coloring schema is summarized in the center of the circle. The “#” symbol indicates genes that include introns. The inner circle shows the GC content of the genome.

The assembled *E. camaldulensis* mitogenome is a single circular sequence of 463,134 bp, with an overall GC content of 45.11% (**Figure 1**). The mitogenome has a quadripartite structure that is characterized by a pair of long (8,986 bp) IR sequences (**Supplementary Figure S1**). Our findings suggest that a circular form, rather than the hypothetical linear structure previously discussed in the case of *E. grandis* (Pinard et al., 2019), is dominant in the mitogenome of *E. camaldulensis*. In our *E. camaldulensis* assembly, we did not find any results that support the linear structure. However, it should be noted that this does not exclude the possibility that the mitogenome contains a head-to-tail concatemer structure *in vivo* (Sloan, 2013).

Annotations were made for 38 conventional protein-coding genes within the single circle model. In addition, 19 tRNA genes and five rRNA genes were observed (**Table 1**). The *E. camaldulensis* mitogenome seems to have lost *rps2* and *rps1* genes (Adams et al., 2002). Although fragmented homologous sequences of *rps19* were detected in the mitogenome, stop codons were also observed in the middle of the gene. Two copies of *5S rRNA* were found, which are also present in the mitogenome of *E. grandis*. Two copies of *18S rRNA* were also annotated on the *E. camaldulensis* mitogenome, which was previously described as a single copy gene in *E. grandis* mitogenome (Pinard et al., 2019). Overall, it appears that the gene content is well conserved between the two *Eucalyptus* mitogenomes.

**Table 1:** Gene composition in the mitogenome of *E. camaldulensis*.

Complex I	nad1 (#)	nad2 (#)	nad3	nad4 (#)
	nad4L	nad5 (#)	nad6	nad7 (#)
	nad9			
Complex II	sdh3	sdh4		
Complex III	cob			
Complex IV	cox1	cox2 (#)	cox3	
Complex V	atp1	atp4	atp6	atp8
	atp9			
Cytochrome C biogenesis	ccmB	ccmC	ccmFc (#)	ccmFn
Ribosomal genes (small subunit)	rpl2 (#)	rpl5	rpl10	rpl16
Ribosomal genes (large subunit)	rps1	rps3 (#)	rps4	rps7
	rps10	rps12	rps13	rps14
Other	matR	mttB		
rRNA	26S rRNA	18S rRNA (x2)	5S rRNA (x2)	
tRNA	tRNA-Asn (x2)	tRNA-Asp	tRNA-Cys	tRNA-Gln
	tRNA-Glu	tRNA-Gly	tRNA-His (pl)	tRNA-Ile (x2)
	tRNA-Lys	tRNA-fMet	tRNA-Met	tRNA-Phe
	tRNA-Pro	tRNA-Ser (x2)	tRNA-Trp (pl)	tRNA-Tyr

The symbol “(#)” labels the genes that contain introns. tRNA genes like the plastid type are indicated by “(pl)”.

### 3.2 Phylogeny of E. camaldulensis

**Figure 2:**
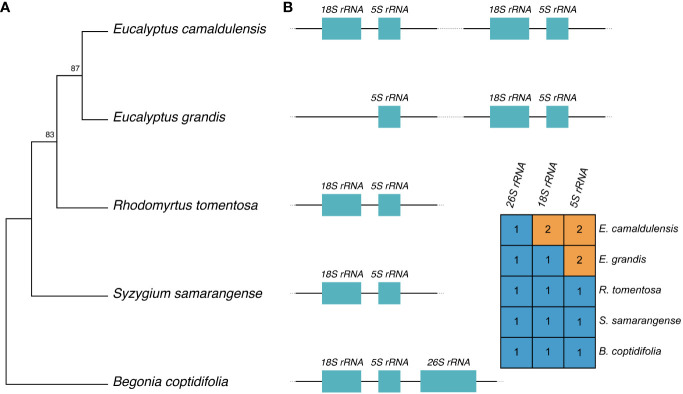
Phylogeny of *E. camaldulensis* and rRNA gene conservation. **(A)** Phylogenetic relationship of *E. camaldulensis*, three *Myrtaceae* plants, and *B. coptidifolia* as an outgroup. The tree is based on the mitogenome sequences of the represented species, and the numbers on the tree nodes represent bootstrap values. **(B)** Relationship between *18S rRNA* and *5S rRNA* genes across five genomes, and their copy numbers are annotated on each mitogenome. The *26S rRNA* gene is also reported; for graphical purposes, only a single representation is used.

To date, there are only three complete mitochondrial sequences from the *Myrtaceae* family being publicly available. For the sake of completeness, we performed a phylogenetic analysis to ascertain the phylogenetic placement of the *E. camaldulensis* mitogenome. The topology of the phylogenetic tree suggests a strong phylogenetic connection between the two eucalypt species, *E. grandis* and *E. camaldulensis* (**Figure 2A**). The tree topology aligns with the phylogenetic tree inferred using mitogenomes by Lu and Li (2024).

The co-transcription and physical proximity of *18S rRNA* and *5S rRNA* in plant mitogenomes have been reported (Chen et al., 2017). Given the topology of the phylogeny, the varying copy numbers of these genes suggest multiple hypotheses for the evolution of these two genes on the mitogenome in the *Myrtaceae* family (**Figure 2B**). The most parsimonious hypothesis is that a single duplication of *5S rRNA* occurred in a common ancestor of the two eucalypts, followed by a duplication of *18S rRNA* in the lineage of *E. camaldulensis*. This hypothesis assumes that there was a duplication event of *18S rRNA* into a proximal physical location of *5S rRNA*. Alternatively, another hypothesis suggests that there were two duplication events of the two genes in a common ancestor, with a subsequent loss of *18S rRNA* that happened in the lineage of *E. grandis*. Estimating the most likely evolution requires additional information regarding the state of the last common ancestor and more complete mitogenomes.

### 3.3 Repeat sequences and major isomeric forms of the mitogenome

**Figure 3:**
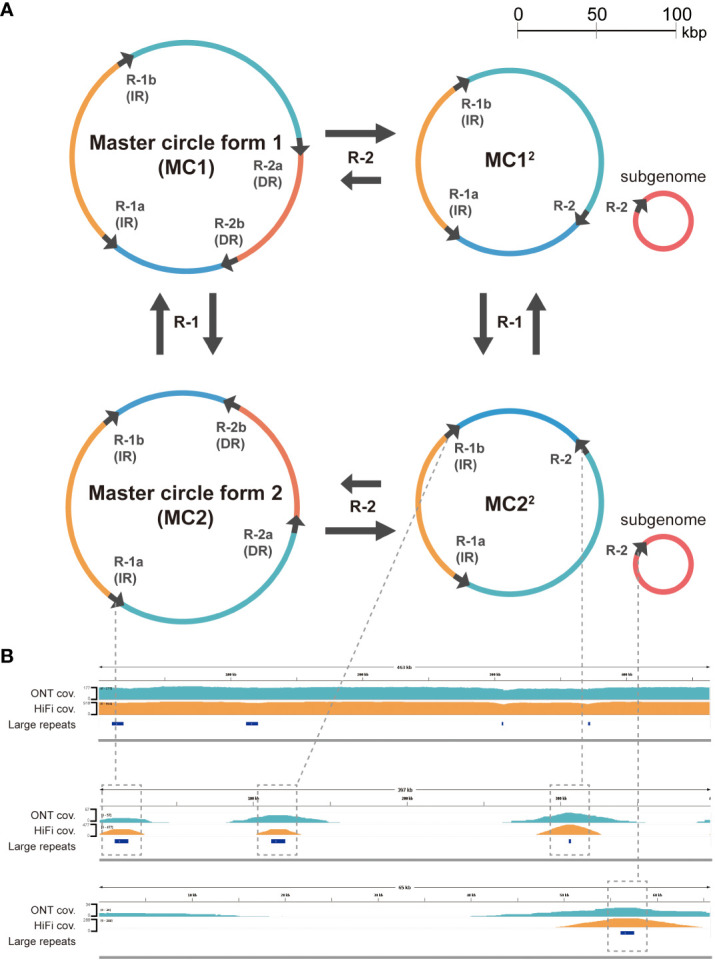
**(A)** Proposed major isomeric forms rearranged by the two large repeats, each greater than 1,000 bp. The large repeats are indicated by black arrows on circles, while the colored arcs represent regions separated by these repeats. The labels adjacent to the arrows on the circles correspond to the repeat names listed in **Supplementary Table S1** and are abbreviated as R-1 and R-2. **(B)** Uniform coverage (cov.) for both ONT and HiFi reads mapped to the assembly (MC1) as visualized by IGV (top). The coverage is slightly lower in the large repeat regions due to the rearrangements mediated by those repeats. These rearrangements are validated by the existence of both ONT and HiFi reads mapped fully only against the rearranged forms (middle and bottom) and not the reference form (top).

The number of detected dispersed repeat sequences was 63, and the repeat size ranged from 50 bp to 9 kb (**Supplementary Table S1**). In the long repeat category (>1,000 bp), we identified pairs of IRs and DRs with lengths of 8,986 and 1,467 bp, respectively. As it has been argued that rearrangement mediated by long repeats is frequent in the plant mitogenomes (Siculella et al., 2001; Sloan, 2013), other possible isomeric forms were investigated by read mapping (see “Materials and methods”). Rearrangements by long repeat sequences are more common than those mediated by middle or short repeat sequences (**Table 2**). In particular, isomeric forms mediated by the pair of the largest IR seem to exist at an equal frequency, similar to other plant mitochondria (Sloan et al., 2010) and chloroplast genomes (Wang and Lanfear, 2019). Rearrangement mediated by the second largest DR pair is also common. The data are consistent with a multipartite mitochondrial genome as a two-circle model (**Figure 3A**). As an attempt to validate these rearrangements mediated by dispersed repeats, we analyzed our assembly using the Oxford Nanopore sequencing platform as an alternative and independent experiment (“Materials and methods”). Using the two independent sequencing technologies, we were able to validate the rearrangements mediated by repeat 1 and repeat 2 (**Figure 3B**). There are also other rearrangements caused by shorter repeats (100 bp to 1 kb), but their frequency is significantly lower (**Table 2**; **Supplementary Table S2**). In fact, 95% of mitochondrial reads mapped against either MC1 or MC2^2^ fully and linearly in both the HiFi and ONT sequencing datasets, and the rest of the mitochondrial reads are mainly related to the minor rearrangements (**Supplementary Table S3**). The HiFi sequencing data and further validation by ONT altogether support four major isomeric forms, including the single circle model (**Figure 3**).

**Table 2:** Rates of the rearrangement mediated by repeat pairs.

Repeat pair	Repeat length	%reference	%alternative
Repeat-1a_b (IR)	8,989	50.98 (104)	49.02 (100)
Repeat-2a_b (DR)	1,467	22.11 (174)	77.89 (613)
Repeat-3a_b (IR)	564	97.74 (822)	2.26 (19)
Repeat-4a_b (IR)	317	98.56 (1095)	1.44 (16)
Repeat-5a_c (IR)	188	98.57 (553)	1.43 (8)
Repeat-5b_c (IR)	188	99.16 (589)	0.84 (5)
Repeat-6a_b (DR)	161	99.53 (1055)	0.47 (5)

Rates were estimated by read mapping. IR and DR stand for inverted repeat and direct repeat, respectively. Numbers in parenthesis in the rate columns indicate the number of supporting reads.

### 3.4 Minor rearrangements hinder *de novo* assembly by haplotype-aware methods

**Figure 4:**
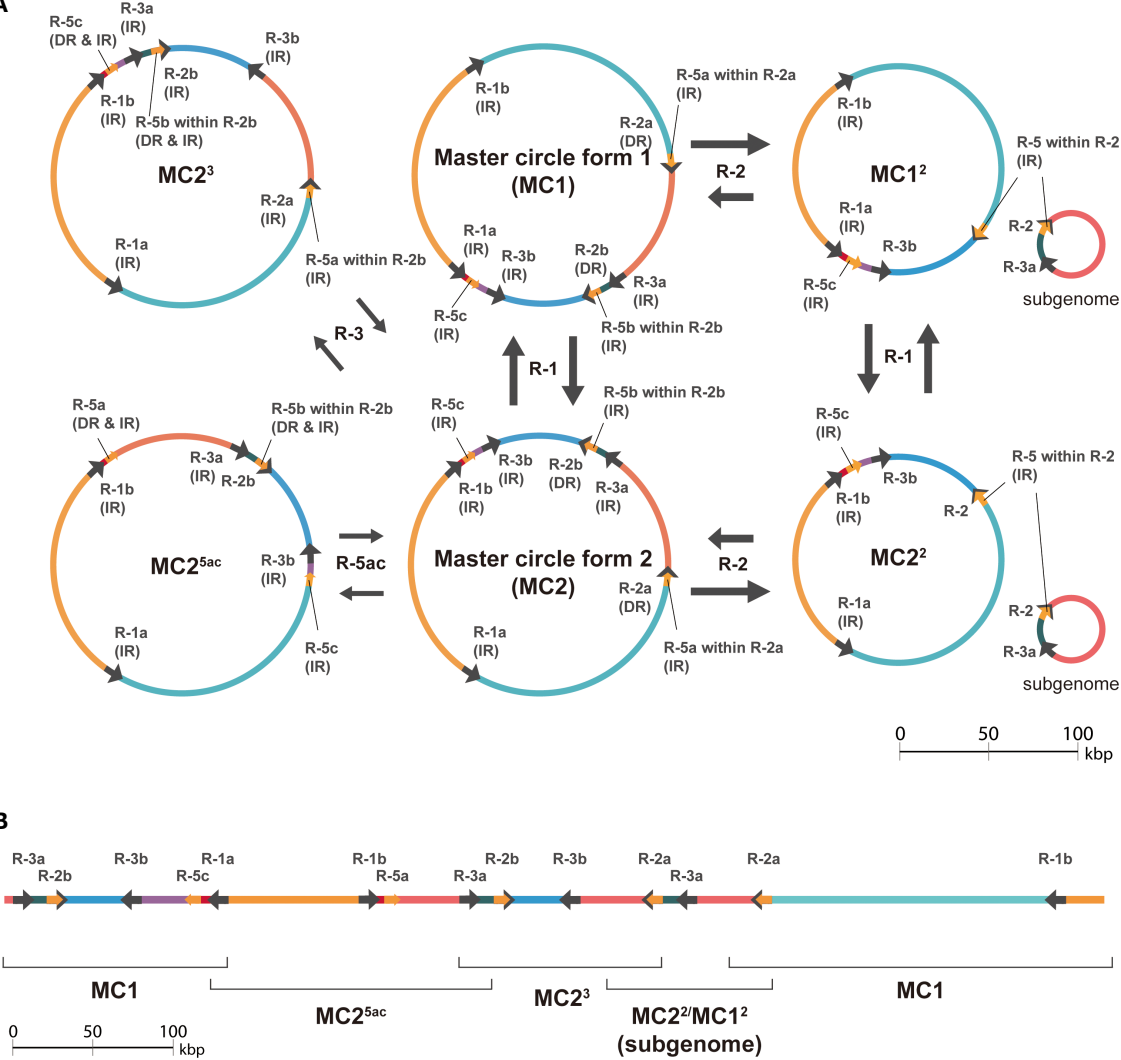
Schematic diagram showing the effects of minor rearrangements mediated by smaller repeat pairs. **(A)** Hypothesized products of major and minor isomeric forms mediated by large (>1,000 bp) repeats-1 and repeat-2, small (< 1,000 bp) repeat-3, and a small and nested repeat-5. The black arrows on circles indicate the repeats, and the colored arcs represent regions separated by the repeats. The labels near the arrows on circles show the repeat names in **Supplementary Table S1**. Repeat-1, repeat-2, repeat-3, and repeat-5 are abbreviated as R-1, R-2, R-3, and R-5 in the figure, respectively. Only relevant forms were extracted, and the full diagram is available as **Supplementary Figure S2**. **(B)** Longest contig (688,965 bp in length) generated by hifiasm which consists of sequences that aligned to hypothesized major isomeric form (MC1) and sequences which do not (MC2^5ac^, MC2^3^, and MC2^2^/MC1^2^). The hypothesized isomeric forms aligned are shown along a schematic line showing the contig. The arrows and colored lines represent repeats and regions separated by the repeats, respectively.

Since the contigs computed by hifiasm only partially matched the major isomeric sequences, we investigated the multiple contigs generated by hifiasm to understand the technical challenges for plant mitogenome assembly. The size of repeat-5 is 188 bp in length, and it is a part of another larger repeat-2 that has a length of 1,467 bp (188 bp of 1,173-th to 1,360-th positions of repeat-2). Repeat-2 is related to one of the major rearrangements (**Figure 3**), and repeat-5 is detected by a 188-bp long sequence far away from the repeat-2 sequences (**Supplementary Table S1**). Rearrangements explained by those small and nested repeat pairs appear to be important despite the low frequency (**Table 2**). Those minor isomeric sequences aligned well to the unique sequences found in the contigs computed by hifiasm, a haplotype-aware assembler (**Figure 4**; **Supplementary Figure S2**). A haplotype-aware assembler such as hifiasm would have concatenated major and minor isomeric forms together as single haplotypes (**Figure 4B**), and this seems to be the cause of multiple contigs.

### 3.5 Large inversion and fragments for the lost copy of *18S rRNA* in *E. grandis* mitogenome

**Figure 5:**
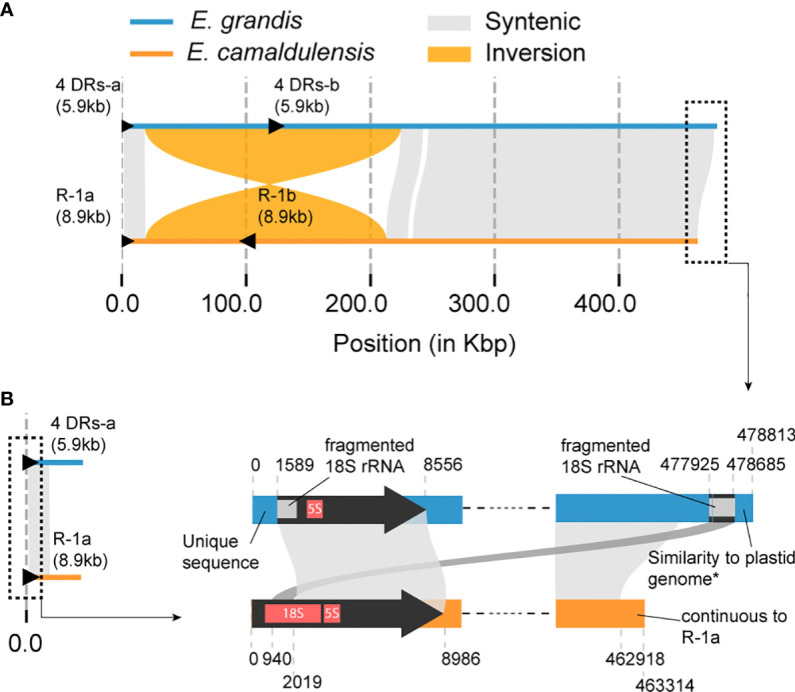
Schematic diagram of the sequence comparison between *E. grandis* and *E. camaldulensis* (MC2) mitogenomes. **(A)** The plot illustrates synteny and structural rearrangements. The largest IR, referred to as “R-1” in **Figures 3**, **4**, is represented by black triangles in the *E. camaldulensis* mitogenome (yellow). Additionally, there are four DR pairs in the *E. grandis* mitogenome (blue), which are homologous to the largest IR, also represented by black triangles. The total size of the four DR sequences and the IR length are shown in parenthesis. **(B)** Detailed sequence comparison in both terminals of two genomes is shown. Similarity to the plastid genome in the very 3′ end of *E. grandis* genome means similarity to some plastid genomes in *Eucalyptus*, which did not include the *E. grandis* plastid genome. Unique sequence in the 5′ end means no significant hit in the nr database of NCBI.

A comparative analysis was performed on the mitogenomes of *E. camaldulensis* and *E. grandis* to understand how synteny is preserved between these two mitogenomes within the same genus. Overall synteny was found to remain intact except for a large inversion observed between positions 18,587 and 224,548 in the *E. grandis* mitogenome (corresponding to positions 19,028 and 212,830 in *E. camaldulensis*) (**Figure 5A**). Despite the fact that synteny is still maintained to some degree, it does not explain a major difference: the hypothetical linear and circular form for them. Due to the absence of other reported mitogenomes in the *Eucalyptus* genus, phylogenetic reconstruction of the ancestral form remains infeasible. However, intriguing fragments with homology to the 5′ terminal of the *E. camaldulensis* genome were detected at both the 5′ and 3′ terminals of the *E. grandis* mitogenome (**Figure 5B**). As indicated earlier, while the *E. camaldulensis* mitogenome encodes two copies of *18S rRNA* (**Table 1**), only one copy is retained in the *E. grandis* mitogenome (**Figure 2B**). Given that this gene is encoded within repeat-1 of the *E. camaldulensis* mitogenome (**Figure 5B**), we can envisage that this gene could serve as a crucial marker for historical tracking without necessitating additional mitogenomes. A sequence comparison using LAST unveiled mutated and fragmented sequences of *18S rRNA* in the *E. grandis* mitogenome (**Figure 5B**). Notably, a fragment resembling a second half of the gene was located at the very 5′ end of the *E. grandis* mitogenome, and a fragment homologous to the first half of the gene was also recognized at the opposite end (3′ end) (**Figure 5B**). It is worth noting that the *E. grandis* mitogenome has unique and non-homologous flanking sequences at both terminals, which is consistent with the genome’s proposed linear structure.

### 3.6 Strong correlations exist between structural variations in the *E. grandis* mitogenome and homologous regions of the *E. camaldulensis* repeat-1

**Figure 6:**
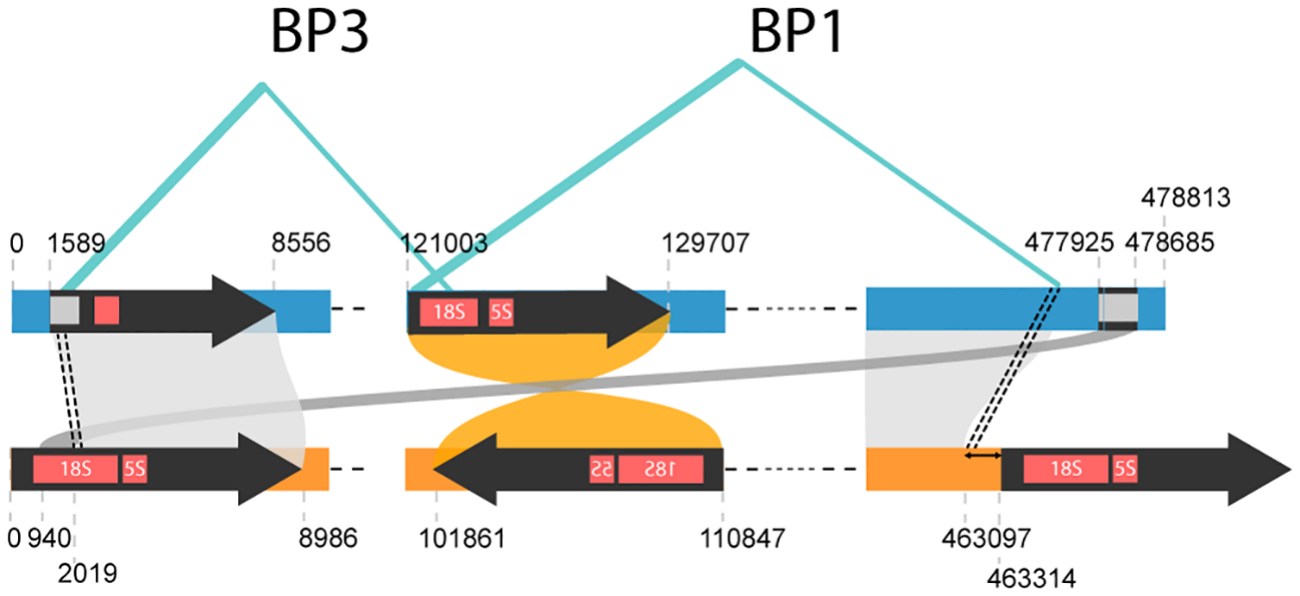
Highly confident structural variations observed in the *E. grandis* mitogenome (blue). We extracted breakpoints for BP1 and BP3 from the original paper. If we map breakpoints for BP3 onto homologous coordinates on *E. camaldulensis* mitogenome (yellow), they pinpoint to a region 2 kb away from the beginning of repeat-1. Similarly, breakpoints for BP1 can be mapped to a very similar point in *E. camaldulensis* mitogenome, around the beginning of repeat-1.

We identified four repeat pairs—DR-1 (1,148 bp), DR-2 (366 bp), DR-3 (4,210 bp), and DR-4 (206 bp)—in *E. grandis* mitogenome (Pinard et al., 2019) as homologous to repeat-1, the largest IR in *E. camaldulensis* (**Figure 5**). The combined length of these four repeat pairs is still shorter than that of repeat-1 (5,930 vs. 8,986 bp), suggesting that a split event may have occurred. On the opposite side of the *E. grandis* genome, a similarity to repeat-1, identified as fragmented *18S rRNA*, is also observed (**Figure 5B**). Assuming that the homologous fragments at both the 5′ and 3′ ends of this mitogenome originated from a single repeat sequence (i.e., repeat-1a), it is possible that either fragment could retain similarity to the homolog of its counterpart, termed repeat-1b. Pinard et al. described three structural variations, termed BP1, BP2, and BP3, with a high degree of confidence (Pinard et al., 2019). Of these variations, BP2 was associated with a rearrangement event triggered by a pair of DRs observed in the *E. grandis* mitogenome. However, no such associations could be made for the other two variations, BP1 and BP3. Notably, the breakpoints of BP3 coincided with two homologous regions 2 kb distant from the start of repeat-1 (**Figure 6**). Furthermore, one breakpoint of BP1 overlapped with a region at the start of repeat-1b, and the other breakpoint coincided with another region homologous to the end of the *E. camaldulensis* mitogenome. Due to its continuous nature (represented as circular form), this end region seamlessly transitions into the start of repeat-1a in the *E. camaldulensis* mitogenome. These structural variants together show a strong correlation (within 250 bp) with respect to the *E. camaldulensis* coordinates (**Figure 6**).

## 4 Discussion

In this study, we present the first and high-quality mitogenome of *E. camaldulensis* which completes the entire genomic assemblies of *E. camaldulensis*. This is also the second report of a mitogenome in the *Eucalyptus* genus. The fact that there are only two mitogenomes compared to the many nuclear genome assemblies from this genus indicates how challenging it is to assemble plant mitogenomes. Genomic features such as low mutation rates and abundant repeats, often involved in homologous recombination, are considered outcomes of DNA repair systems, particularly those addressing double-strand breaks (DSBs) (Christensen, 2013). In this context, repeat-mediated rearrangements are viewed as repair system errors. This aligns with common features observed in plant mitogenomes, especially those rearrangements facilitated by small repeat sequences. Larger repeat sequences (i.e., repeats longer than 1,000 bp) have been found to induce structural changes or isomerization in plant mitogenomes (Sloan et al., 2010; Wynn and Christensen, 2019).

Our results indicate that the rate of rearrangement by the largest repeat remains steady at around 50%; however, the rate for the second largest repeat is more unbalanced (**Table 2**; **Supplementary Table S2**). Based on these findings, we propose four primary isomeric forms comprising both single circle structures and multi-circular sub-genomic structures (**Figure 3A**).

HiFi reads, which we used in the assembly of the mitogenome, confirmed the existence of the significant rearrangements mediated by the largest and the second largest repeats (**Figure 3B**). In fact, even with accurate long reads, the mitogenome assembly can still be challenging in repetitive regions (Chen et al., 2021). Therefore, we manually analyzed repeat regions highlighting those reads that span the entire repeat as well as flanking sequences to rule out any potential errors introduced by repeat units on the assembly graph (**Supplementary Figures S3, S4**).

Additionally, we further validated our results using an ONT sequencing dataset that was not used in the genome assembly. Overall, both HiFi and ONT reads aligned to the assembled sequences of the major isoforms, validating the assembly (**Figure 3B**; **Supplementary Figures S3, S4**). These experiments and analyses support the existence of multiple isoforms of the genome and their respective sequences. This characteristic of plant mitogenomes has implications for phylogenetic analysis, especially among closely related species. While low mutation rates in protein coding genes and high variation in non-coding regions pose technical challenges in phylogenetic analysis, sequence alignment that considers rearrangement is helpful in overcoming these challenges.

A nested relationship was observed in the genome as one repeat sequence, repeat-5, is part of a larger sequence, termed repeat-2. While nested pair rearrangements appear to be infrequent, such complex repeat pairs hampered assembly by haplotype-aware methods, presumably due to the methods’ sensitivity. Haplotype-aware assemblers aim to keep one haplotype contiguous (Cheng et al., 2021), which is not always ideal for organelle genome assemblies. Indeed an approach that takes into account the characteristics of plant organelle genomes would be better suited to reconstructing major forms of plant mitochondrial genomes that may have undergone complex rearrangements with identical sequences.

Even if major forms could be partially detected by a conventional approach, a *post-hoc* analysis was still required to obtain the full picture of mitogenome dynamism. Infrequent nested pair rearrangements need complex structures, even when the repeat graph format is represented. An example of this complexity is a “triple-fork” structure to represent rearrangements mediated by nested repeat pairs, which is more intricate than the recently suggested “double-fork” structure (Fischer et al., 2022). This observation shows that more theoretical progress is needed in this field.

In regard to the evolution of *18S* and *5S rRNA* genes, the presence of mutated *18S rRNA* in the mitogenome of *E. grandis* supports that there was another copy of *18S rRNA* and the loss event of that copy. It is likely that the last common ancestor of *E. grandis* and *E. camaldulensis* possessed two copies of *18S rRNA* in the mitogenome. An alternative scenario would involve two independent duplications of the genes after the divergence to the ancestors of *E. camaldulensis* and *E. grandis*. However, this scenario is less likely because it assumes two duplication events of the gene into a similar physical location on each genome, i.e., next to *5S rRNA*. It is worth noting that the mitogenomes of *S. samarangense*, *R. tomentosa*, and *B. coptidifolia* are annotated to contain only one copy of *18S rRNA* and *5S rRNA*. It is likely that the duplication of a relatively long sequence containing both *18S rRNA* and *5S rRNA* genes, rather than the duplication of single genes, occurred during the divergence to the *Eucalyptus* genus in the *Myrtaceae* family, followed by the loss of *18S rRNA* gene in the lineage of *E. grandis* along with the splitting of the repeat sequence (**Figure 2**).

If DSBs are common in the plant mitochondria, failure in DNA repair mechanisms may be inherited and maintained over evolutionary time. If DSB is a rare condition, failure in DNA repair mechanisms would have less impact; however, it is important to note that rearrangements can also lead to the development of novel biological functions. One possible example is cytoplasmic male sterility, a phenomenon in plants that prevents them from producing viable pollen and has been linked to mitochondrial genome rearrangements (Mower et al., 2012). DSBs could be the reason for the discrepancy between the mitogenomes of *E. camaldulensis* and *E. grandis*. As discussed above, the presence of a mutated and fragmented copy of *18S rRNA* in the mitogenome of *E. grandis* suggests that the last common ancestral mitogenome of both *E. grandis* and *E, camaldulensis* should have contained two copies of *18S rRNA* similar to the mitochondria of *E. camaldulensis*. Following this suggestion, a DSB event could have occurred in between a region equivalent to repeat-1a. This is also supported by the non-homologous sequences at both ends of *E. grandis* mitogenome (**Figure 5B**). The hypothetical linear form of the *E. grandis* genome is consistent with the flanking sequences at both 5′ and 3′ ends, which are dissimilar to those of *E. camaldulensis* and any other mitogenome. The 5′ flanking sequence is quite unique, and we were unable to detect any significant matches in the NCBI nucleotide database. There is instead a similarity between the 3′ flanking sequence and the chloroplast genomes of other plant species. Unfortunately, we lack data to explain the reason, and more mitogenomes and further analysis will be required in the future.

One interesting nature of repeat sequences is their plasticity. Any subsequences in a repeat sequence can also generate a new repeat pair if the equivalent subsequence appears in another location. This plasticity feature has been detected in our study as nested repeat sequences. Rearrangements mediated by nested repeat pairs were also observed in the longest contig computed by hifiasm. Conventional genome assemblers are not designed to resolve all variations caused by complex repeats (Fischer et al., 2022). Multiple algorithms should be applied to complete both nuclear and organelle genome assemblies, at least in plant genome projects.

The other feature of repeat plasticity is high separability. If a repeat sequence of a pair is cut *n* times, *n+1* subsequences should keep similarity to the counterparts in the other sequence of the pair. This could lead to new *n+1* pairs of repeat sequences in the following generations if inherited. Although it was not obvious in the primary structure of the *E. grandis* mitogenome, two high-confidence structural variations seem to be two recent pairs of repeats generated by a single DSB event (**Figure 6**). Even breakage of a repeat would not have lost its repeat characteristic.

## 5 Conclusion

In this study, we presented the first and high-quality mitogenome of *E. camaldulensis*. Although the mitogenome is much smaller than the nuclear genome, its nature in plant species could hinder genome assembly. Rearrangements mediated by dispersed repeat can lead to fragmentation in the assembly graph, having resulted in a lower number of available sequences than the nuclear assembly. A comparison of the mitogenomes of *E. camaldulensis* and *E. grandis* revealed a unique alteration of the repeat sequences at the evolutionary scale. Repeat sequences exhibit a high level of plasticity, which may contribute to the diversity and evolution of plant mitochondria. The presented mitogenome will serve as a reference for further analyses of other eucalypts.

## Data availability statement

The complete mitogenome sequence of *E. camaldulensis* has been submitted to GenBank with the accession number OR763353. The ONT sequencing data utilized for the assembly validation is available under NCBI BioProject ID PRJNA737587. 

## Author contributions

YF: Writing – original draft, Visualization, Funding acquisition, Formal Analysis, Conceptualization. PD: Writing – review & editing, Resources, Investigation. SB: Validation, Writing – review & editing, Data curation. KC: Writing – review & editing, Investigation, Data curation. AP: Writing – review & editing, Investigation, Data curation. M-SC: Writing – review & editing, Visualization, Resources. LE: Writing – original draft, Formal Analysis, Conceptualization.

## References

[B1] Adams K. L. Qiu Y.-L. Stoutemyer M. Palmer J. D. (2002). Punctuated evolution of mitochondrial gene content: high and variable rates of mitochondrial gene loss and transfer to the nucleus during angiosperm evolution. Proc. Natl. Acad. Sci. United States America 99, 9905–9125. doi: 10.1073/pnas.042694899 PMC126597 12119382

[B2] Altschul S. F. Madden T. L. Schäffer A. A. Zhang J. Zhang Z. Miller W.. (1997). Gapped BLAST and PSI-BLAST: A new generation of protein database search programs. Nucleic Acids Res. 25, 3389–3402. doi: 10.1093/nar/25.17.3389 9254694 PMC146917

[B3] Alverson A. J. Wei X. Rice D. W. Stern D. B. Barry K. Palmer J. D. (2010). Insights into the evolution of mitochondrial genome size from complete sequences of citrullus lanatus and cucurbita pepo (Cucurbitaceae). Mol. Biol. Evol. 27, 1436–1485. doi: 10.1093/molbev/msq029 20118192 PMC2877997

[B4] Arrieta-Montiel M. P. Shedge V. Davila J. Christensen A. C. Mackenzie S. A. (2009). Diversity of the arabidopsis mitochondrial genome occurs via Nuclear-Controlled Recombination Activity. Genet. 183, 1261–1685. doi: 10.1534/genetics.109.108514 PMC2787419 19822729

[B5] Butcher P. A. Otero A. McDonald M. W. Moran G. F. (2002). Nuclear RFLP variation in eucalyptus camaldulensis dehnh. from Northern Australia. Heredity 88, 402–412. doi: 10.1038/sj.hdy.6800074 11986878

[B6] Chan P. P. Lin B. Y. Mak A. J. Lowe T. M. (2021). TRNAscan-SE 2.0: improved detection and functional classification of transfer RNA genes. Nucleic Acids Res. 49, 9077–9965. doi: 10.1093/nar/gkab688 34417604 PMC8450103

[B7] Chen Y. Zhang Y. Wang A. Y. Gao M. Chong Z. (2021). Accurate long-read de novo assembly evaluation with inspector. Genome Biol. 22, 3125. doi: 10.1186/s13059-021-02527-4 PMC8590762 34775997

[B8] Chen Z. Zhao N. Li S. Grover C. E. Nie H. Wendel J. F.. (2017). Plant mitochondrial genome evolution and cytoplasmic male sterility. Crit. Rev. Plant Sci. 36, 55–695. doi: 10.1080/07352689.2017.1327762

[B9] Cheng H. Concepcion G. T. Feng X. Zhang H. Li H. (2021). Haplotype-resolved de novo assembly using phased assembly graphs with hifiasm. Nat. Methods 18, 170–755. doi: 10.1038/s41592-020-01056-5 33526886 PMC7961889

[B10] Christensen A. C. (2013). Plant mitochondrial genome evolution can be explained by DNA repair mechanisms. Genome Biol. Evol. 5, 1079–1086. doi: 10.1093/gbe/evt069 23645599 PMC3698917

[B11] Cole L. W. Guo W. Mower J. P. Palmer J. D. (2018). High and variable rates of repeat-mediated mitochondrial genome rearrangement in a genus of plants. Mol. Biol. Evol. 35, 2773–2855. doi: 10.1093/molbev/msy176 30202905

[B12] Darling A. E. Mau B. Perna N. T. (2010). ProgressiveMauve: multiple genome alignment with gene gain, loss and rearrangement. PloS One 5, e111475. doi: 10.1371/journal.pone.0011147 PMC2892488 20593022

[B13] Driguez P. Bougouffa S. Carty K. Putra A. Jabbari K. Reddy M.. (2021). LeafGo: leaf to genome, a quick workflow to produce high-quality de novo plant genomes using long-read sequencing technology. Genome Biol. 22, 2565. doi: 10.1186/s13059-021-02475-z PMC8414726 34479618

[B14] Fischer A. Dotzek J. Walther D. Greiner S. (2022). Graph-based models of the oenothera mitochondrial genome capture the enormous complexity of higher plant mitochondrial DNA organization. NAR Genomics Bioinf. 4, lqac027. doi: 10.1093/nargab/lqac027 PMC8969700 35372837

[B15] Fukasawa Y. Tsuji J. Fu S. C. Tomii K. Horton P. (2015). MitoFates: improved prediction of mitochondrial targeting sequences and their cleavage sites. Mol. Cell. Proteomics. 14, 1113–1126. doi: 10.1074/mcp.M114.043083 25670805 PMC4390256

[B16] Goel M. Sun H. Jiao W.-B. Schneeberger K. (2019). SyRI: finding genomic rearrangements and local sequence differences from whole-genome assemblies. Genome Biol. 20, 2775. doi: 10.1186/s13059-019-1911-0 PMC6913012 31842948

[B17] Greiner S. Lehwark P. Bock R. (2019). OrganellarGenomeDRAW (OGDRAW) version 1.3.1: expanded toolkit for the graphical visualization of organellar genomes. Nucleic Acids Res. 47, W59–W64. doi: 10.1093/nar/gkz238 30949694 PMC6602502

[B18] Kiełbasa S. M. Wan R. Sato K. Horton P. Frith M. C. (2011). Adaptive seeds tame genomic sequence comparison. Genome Res. 21, 487–935. doi: 10.1101/gr.113985.110 21209072 PMC3044862

[B19] Kolmogorov M. Yuan J. Lin Yu Pevzner P. A. (2019). Assembly of long, error-prone reads using repeat graphs. Nat. Biotechnol. 37, 540–465. doi: 10.1038/s41587-019-0072-8 30936562

[B20] Li H. (2018). Minimap2: pairwise alignment for nucleotide sequences. Bioinformatics 34, 3094–3100. doi: 10.1093/bioinformatics/bty191 29750242 PMC6137996

[B21] Lötter A. Duong T. A. Candotti J. Mizrachi E. Wegrzyn J. L. Myburg A. A. (2022). Haplogenome assembly reveals structural variation in eucalyptus interspecific hybrids. GigaScience 12, giad064. doi: 10.1093/gigascience/giad064 37632754 PMC10460159

[B22] Lu G. Li Q. (2024). Complete mitochondrial genome of syzygium samarangense reveals genomic recombination, gene transfer, and RNA editing events. Front. Plant Sci. 14. doi: 10.3389/fpls.2023.1301164 PMC10803518 38264024

[B23] Mower J. P. Case A. L. Floro E. R. Willis J. H. (2012). Evidence against equimolarity of large repeat arrangements and a predominant master circle structure of the mitochondrial genome from a monkeyflower (Mimulus guttatus) lineage with cryptic CMS. Genome Biol. Evol. 4, 670–865. doi: 10.1093/gbe/evs042 22534162 PMC3381676

[B24] Myburg A. A. Grattapaglia D. Tuskan G. A. Hellsten U. Hayes R. D. Grimwood J.. (2014). The genome of eucalyptus grandis. Nature 510, 356–362. doi: 10.1038/nature13308 24919147

[B25] Pinard D. Myburg A. A. Mizrachi E. (2019). The plastid and mitochondrial genomes of eucalyptus grandis. BMC Genomics 20, 1325. doi: 10.1186/s12864-019-5444-4 PMC6373115 30760198

[B26] Siculella L. Damiano F. Cortese M. R. Dassisti E. Rainaldi G. Gallerani R.. (2001). Gene content and organization of the oat mitochondrial genome. TAG. Theor. Appl. Genet. Theoretische Und Angewandte Genetik 103, 359–365. doi: 10.1007/s001220100568

[B27] Sloan D. B. (2013). One ring to rule them all? Genome sequencing provides new insights into the ‘master circle’ Model of plant mitochondrial DNA structure. New Phytol. 200, 978–985. doi: 10.1111/nph.12395 24712049

[B28] Sloan D. B. Alverson A. J. Storchová H. Palmer J. D. Taylor D. R. (2010). Extensive loss of translational genes in the structurally dynamic mitochondrial genome of the angiosperm silene latifolia. BMC Evol. Biol. 274, 1–15. doi: 10.1186/1471-2148-10-274 PMC2942850 20831793

[B29] Sun M. Zhang M. Chen X. Liu Y. Liu B. Li J.. (2022). Rearrangement and domestication as drivers of rosaceae mitogenome plasticity. BMC Biol. 20, 1815. doi: 10.1186/s12915-022-01383-3 PMC9392253 35986276

[B30] Tamura K. Stecher G. Kumar S. (2021). MEGA11: molecular evolutionary genetics analysis version 11. Mol. Biol. Evol. 38, 3022–3275. doi: 10.1093/molbev/msab120 33892491 PMC8233496

[B31] Wang W. Das A. Kainer D. Schalamun M. Morales-Suarez A. Schwessinger B.. (2020). The draft nuclear genome assembly of eucalyptus pauciflora: A pipeline for comparing de novo assemblies. GigaScience 9, giz160. doi: 10.1093/gigascience/giz160 31895413 PMC6939829

[B32] Wang W. Lanfear R. (2019). Long-reads reveal that the chloroplast genome exists in two distinct versions in most plants. Genome Biol. Evol. 11, 3372–3815. doi: 10.1093/gbe/evz256 31750905 PMC7145664

[B33] Wu Z. Waneka G. Broz A. K. King C. R. Sloan D. B. (2020). MSH1 is required for maintenance of the low mutation rates in plant mitochondrial and plastid genomes. Proc. Natl. Acad. Sci. U. S. A. 117, 16448–16555. doi: 10.1073/pnas.2001998117 32601224 PMC7368333

[B34] Wynn E. L. Christensen A. C. (2019). Repeats of unusual size in plant mitochondrial genomes: identification, incidence and evolution. G3: Genes Genom. Genet. 9, 549–595. doi: 10.1534/g3.118.200948 PMC6385970 30563833

