# Supplementary material for: Plasticity of repetitive sequences demonstrated by the complete mitochondrial genome of Eucalyptus camaldulensis
Source: Front Plant Sci. 2024 Mar 27;15:1339594. doi: 10.3389/fpls.2024.1339594 (PMC11005031; doi:10.3389/fpls.2024.1339594)
Supplement: Supplementary file 1 [file DataSheet_1.docx]

***Supplementary Material***

# Supplementary Figures and Tables

## Supplementary Figures


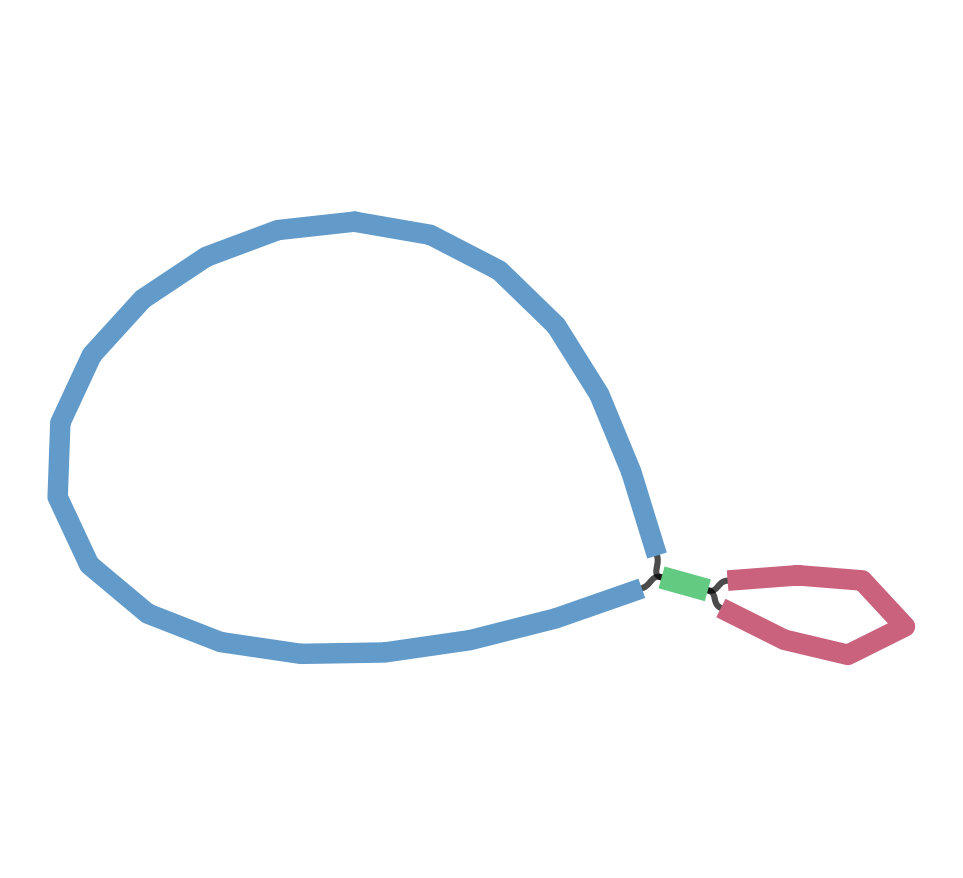


**Supplementary Figure 1**: Graph representation of the contig for *E. camaldulensis* mitogenome.


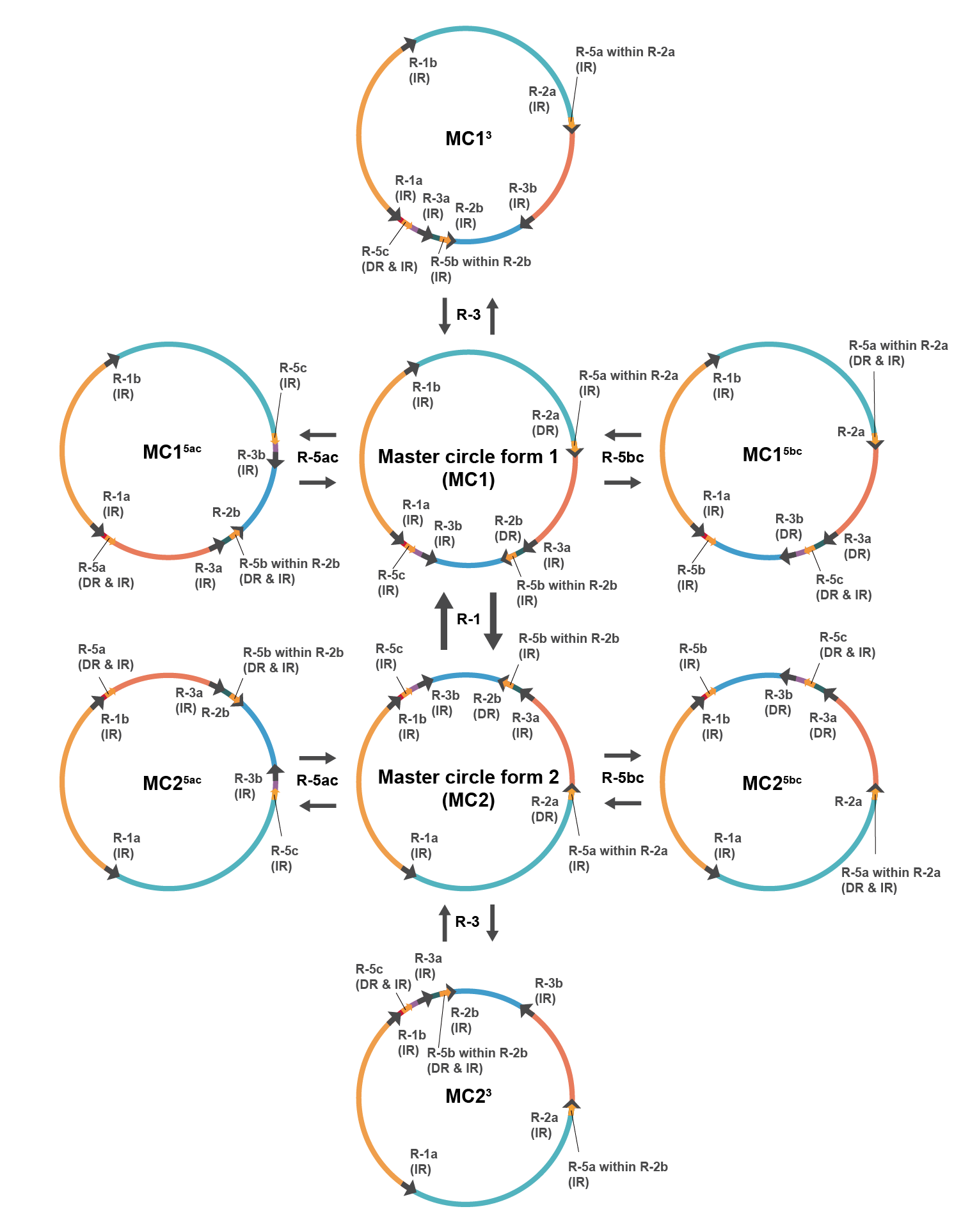


**Supplementary Figure** **2**: Schematic diagram showing effects of minor rearrangements mediated by smaller repeats. The hypothetical products of isomeric forms mediated by large (>1,000 bp) repeat-1, small (< 1,000 bp) repeat-3, and a small and nested repeat-5. Black arrows on circles indicate the repeats, and colored arcs represent regions separated by the repeats. The labels near the arrows on circles show repeat names in Table S1 and are abbreviated as R-1, R-3, and R-5, respectively.


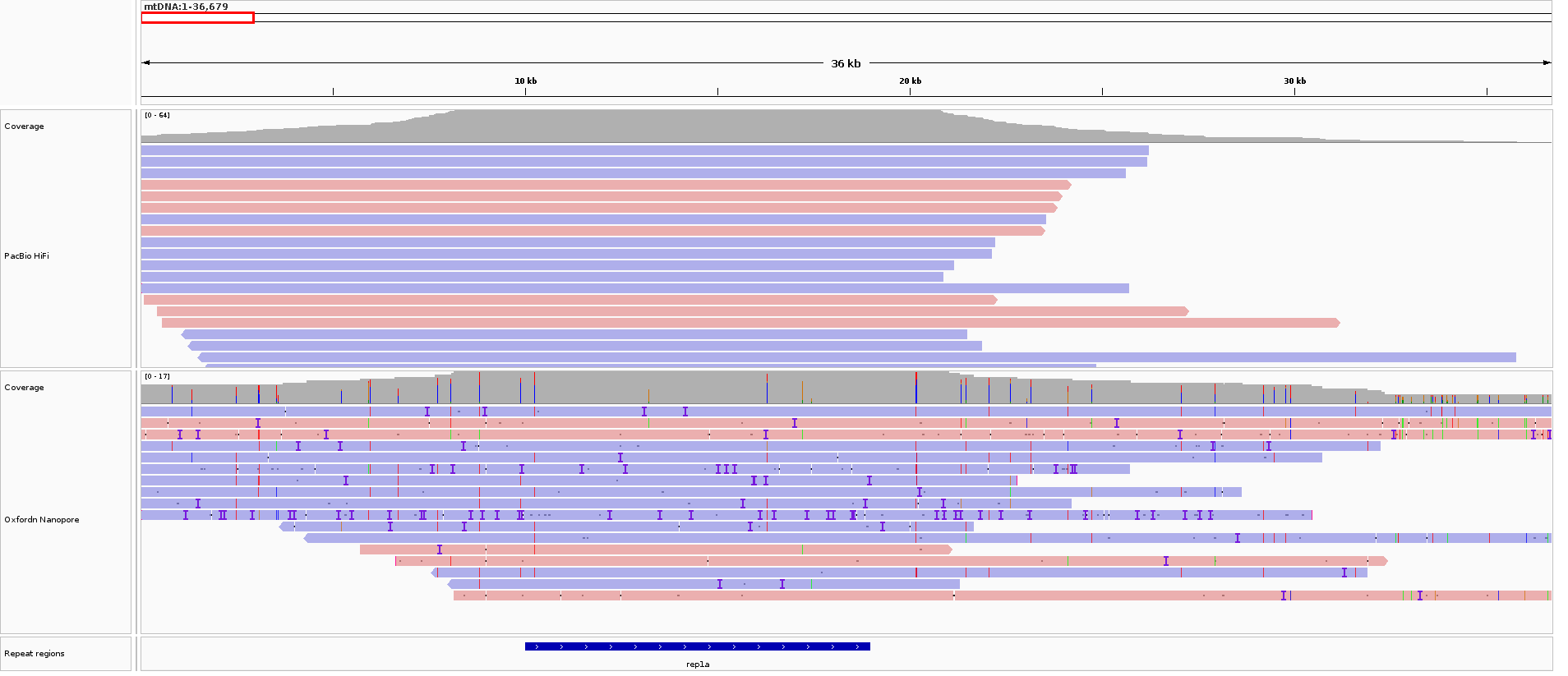

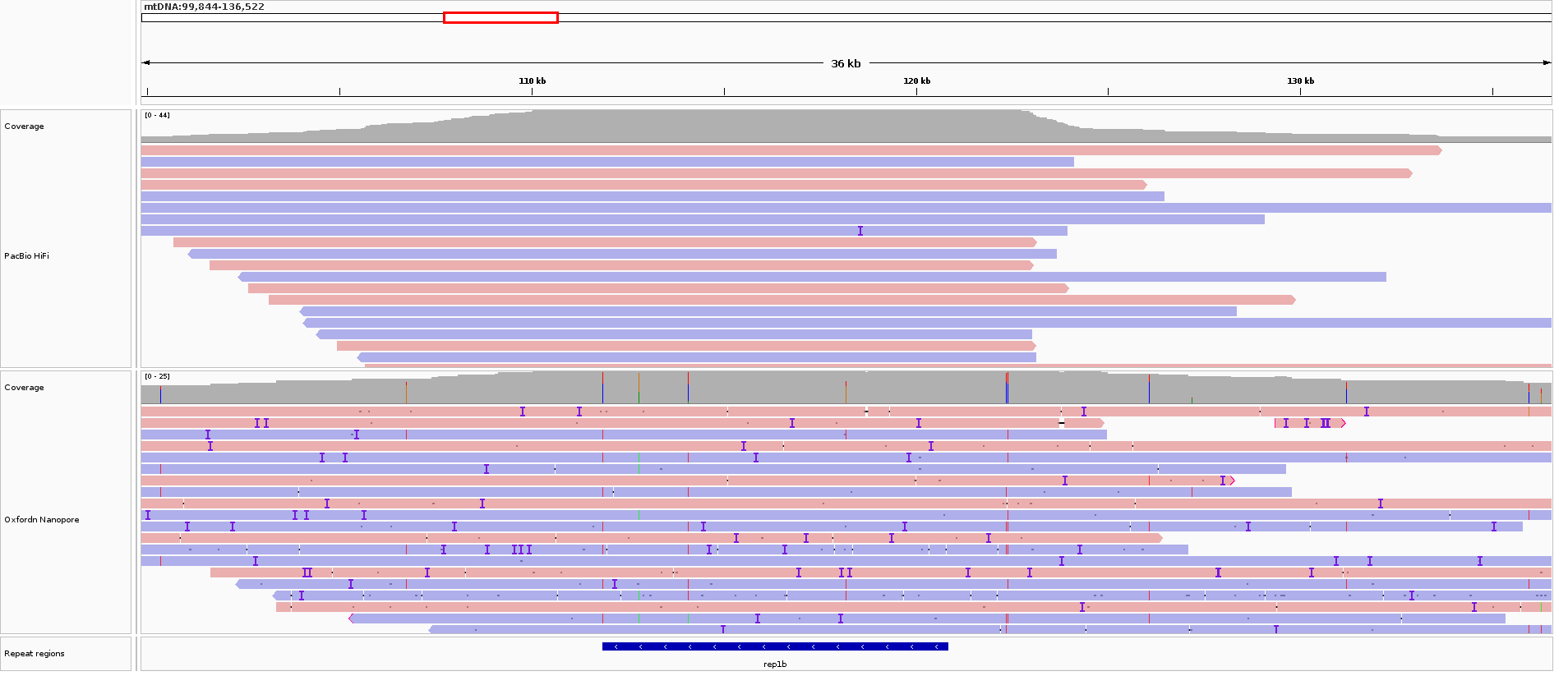

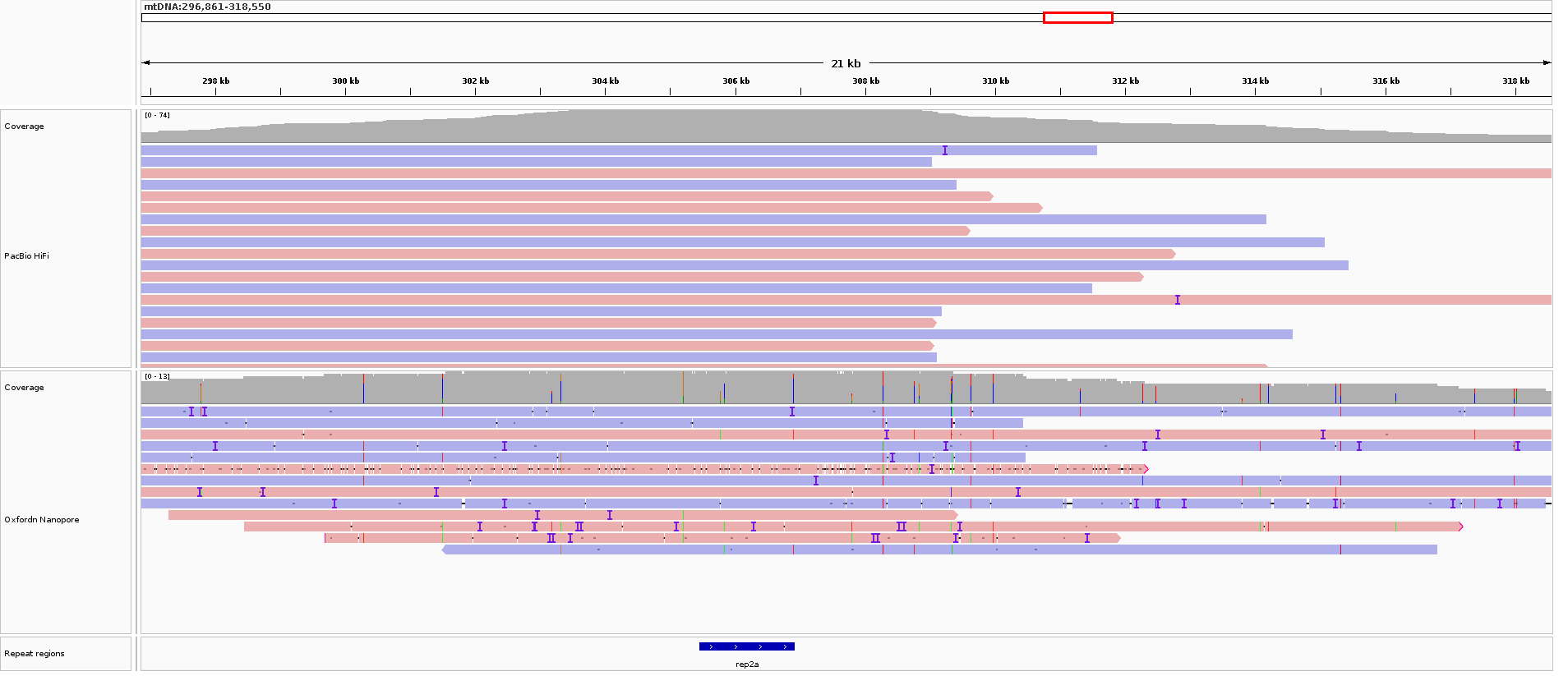

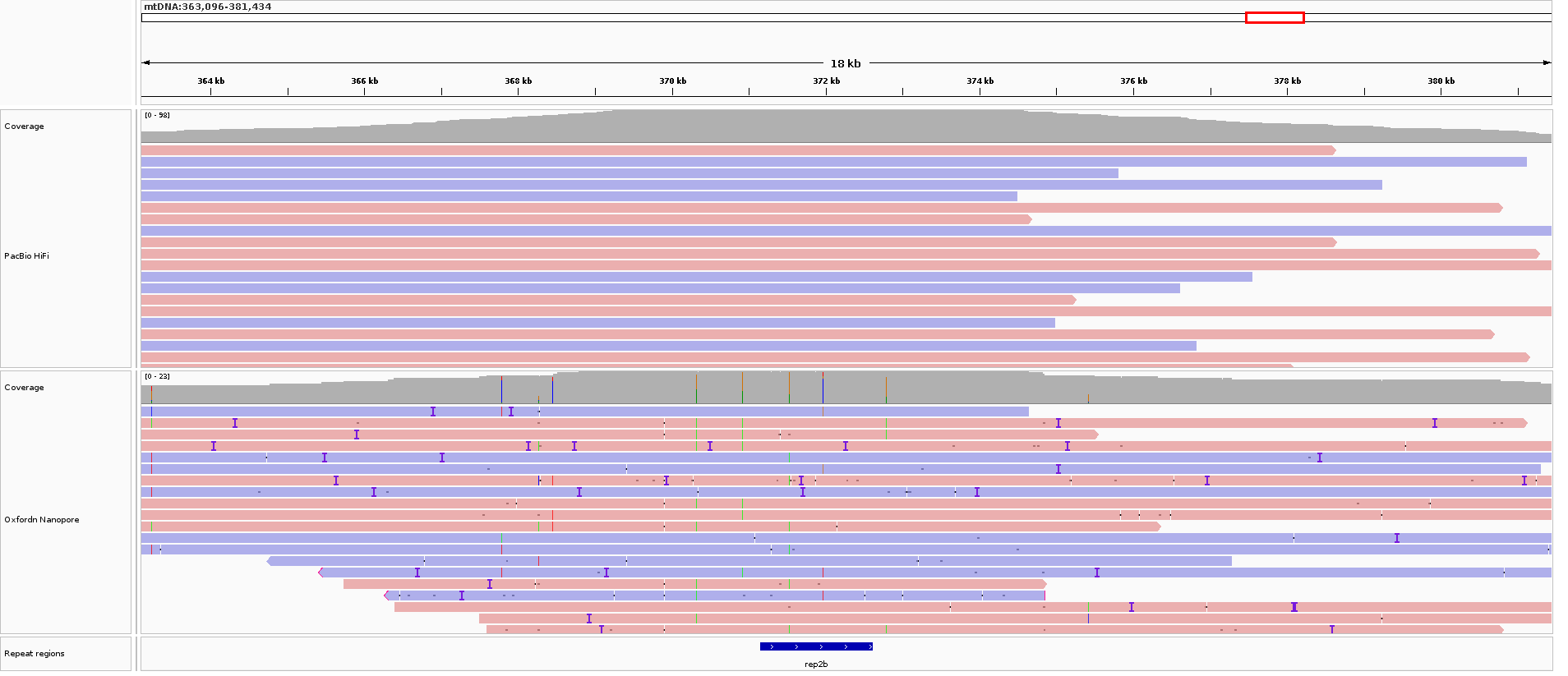


**Supplementary Figure** **3:** Assembled sequences in the four large repeat regions were manually confirmed using reads spanning repeat and surrounding flanking sequences as visualized by IGV. The alignments for repeat-1a, repeat-1b, repeat-2a, and repeat-2b are shown sequentially from top to bottom. The top and bottom panel show alignments of HiFi and ONT reads against MC1, respectively.


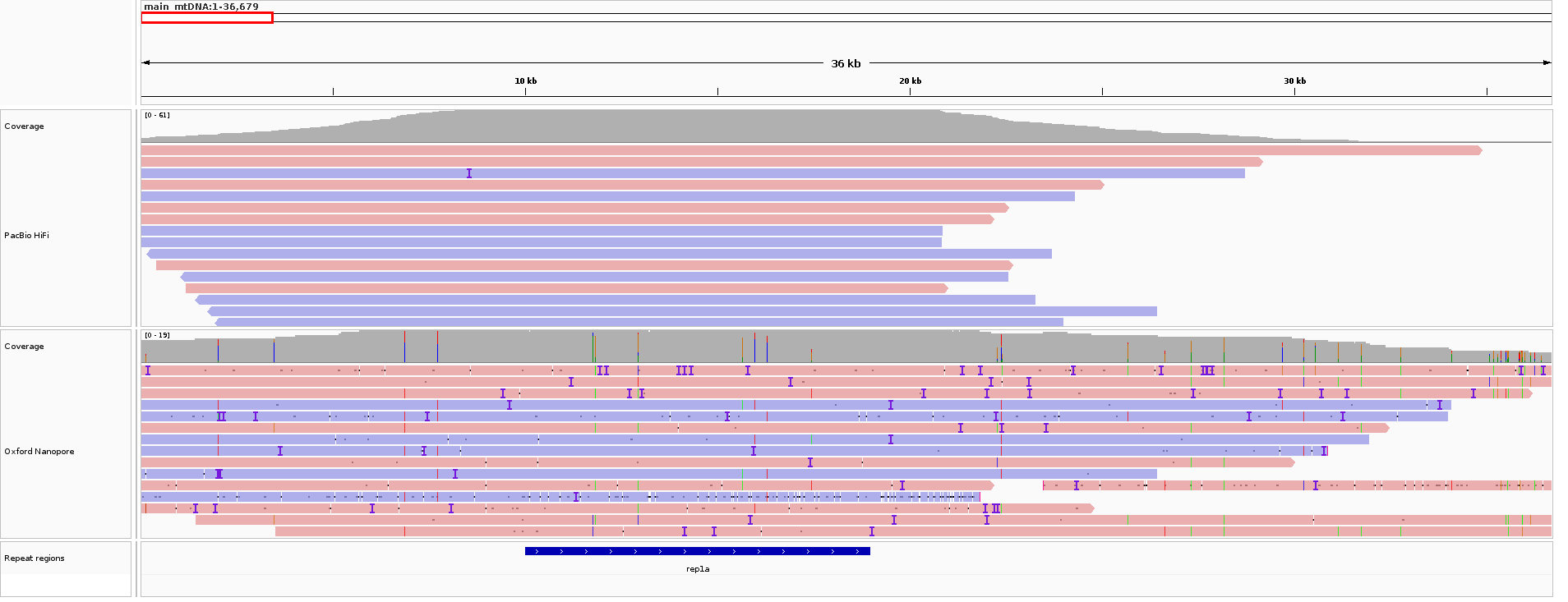

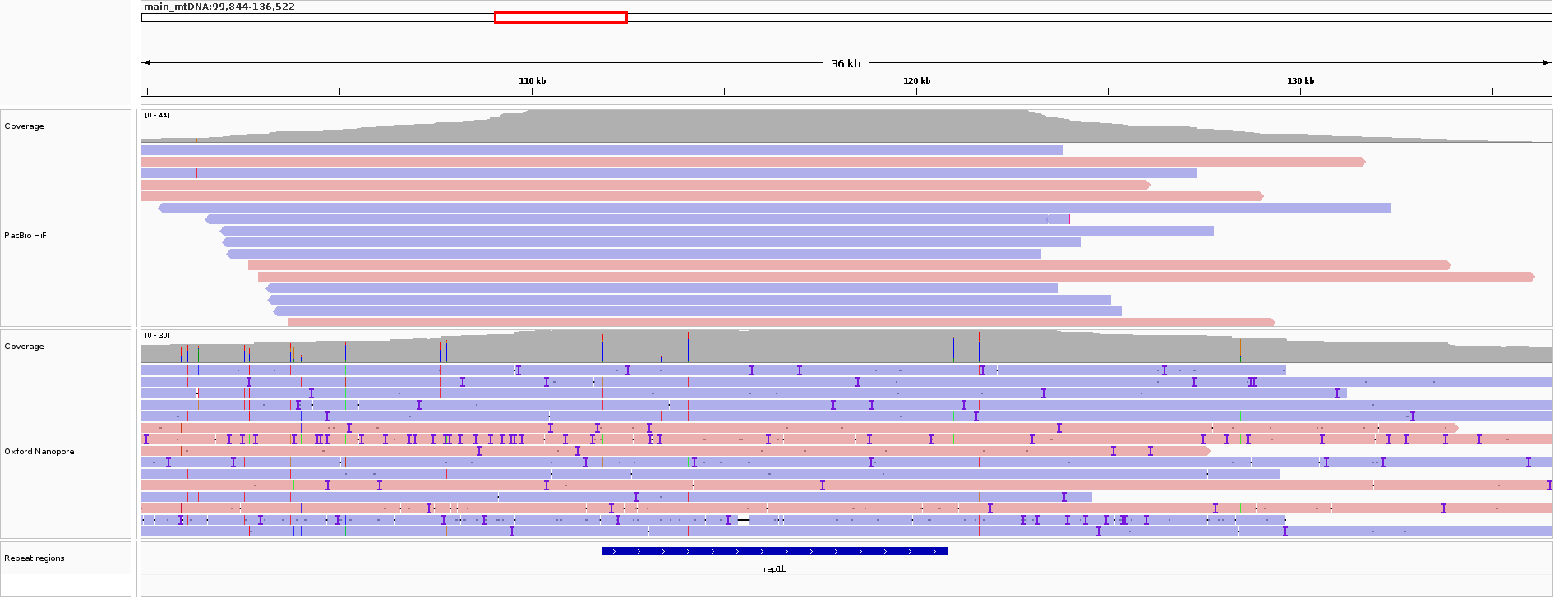

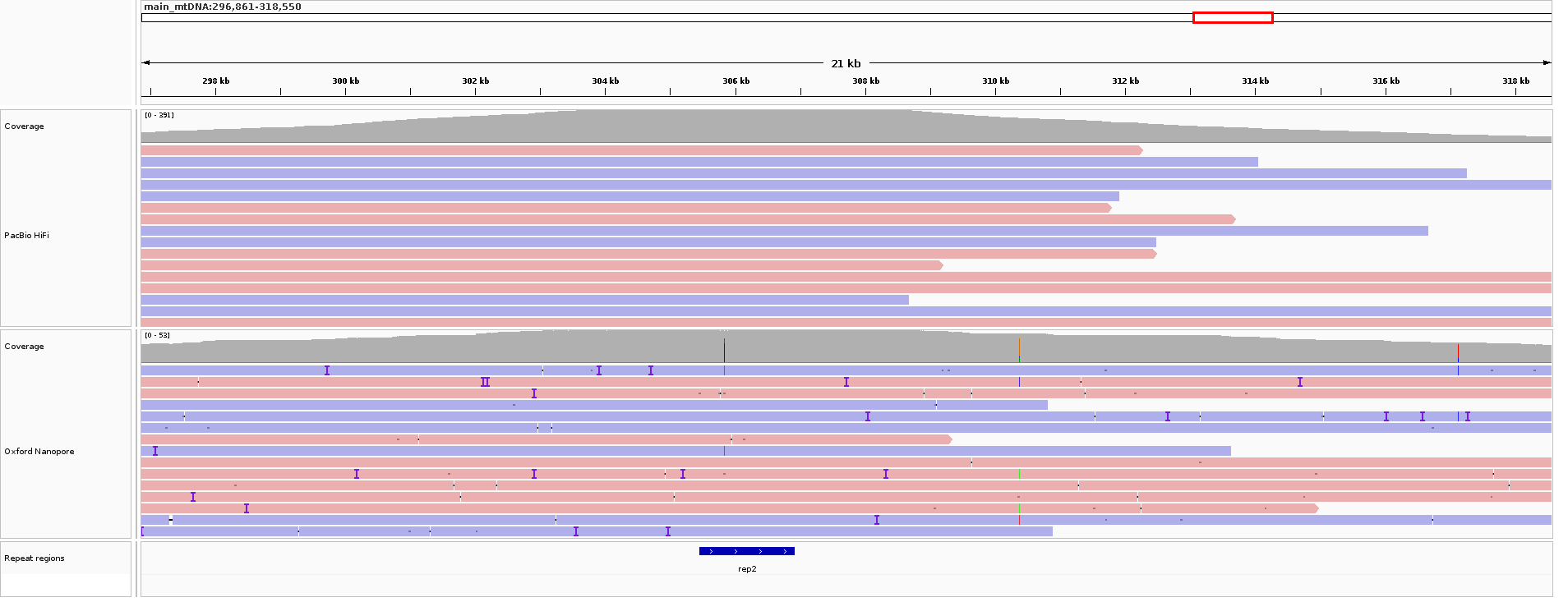

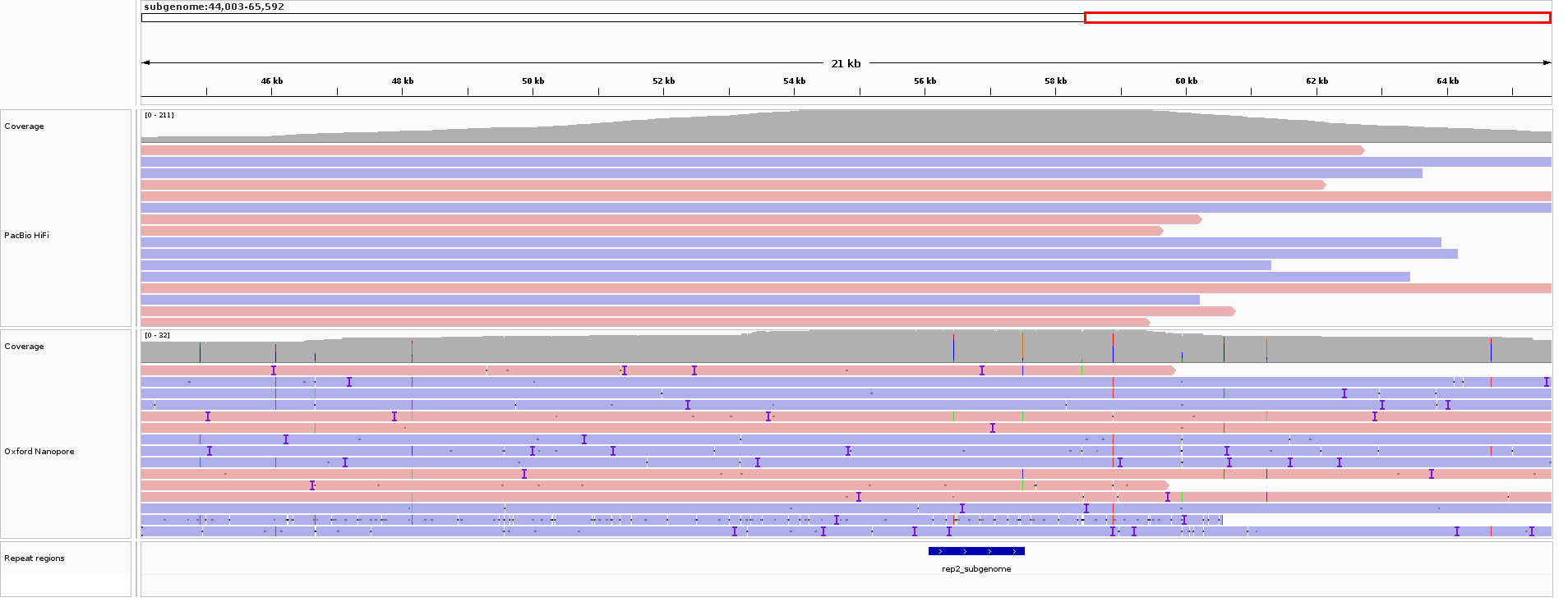


**Supplementary Figure** **4:** Assembled sequences in the four large repeat regions were manually confirmed using reads spanning repeat and surrounding flanking sequences as visualized by IGV. The alignments for repeat-1a, repeat-1b, repeat-2, and repeat-2 in the subgenome are shown sequentially from top to bottom. The top and bottom panel show alignments of HiFi and ONT reads against MC2^2^, respectively.

## Supplementary Tables

**Supplementary Table 1**: Repeat pairs recognized by ROUSfinder. Order in each pair was decided by the tool.

| **ID** | **length** | **coordinates** | | **strand** | **remark** |
| --- | --- | --- | --- | --- | --- |
| Repeat-1a | 8989 | 1 | 8989 | plus |  |
| Repeat-1b | 8989 | 110847 | 101859 | minus |  |
| Repeat-2a | 1467 | 295460 | 296926 | plus |  |
| Repeat-2b | 1467 | 361152 | 362618 | plus |  |
| Repeat-3a | 564 | 343377 | 343940 | plus |  |
| Repeat-3b | 564 | 405854 | 405291 | minus |  |
| Repeat-4a | 317 | 66758 | 67074 | plus |  |
| Repeat-4b | 317 | 373236 | 372920 | minus |  |
| Repeat-5a | 188 | 296632 | 296819 | plus | nested in Repeat-2a |
| Repeat-5b | 188 | 362324 | 362511 | plus | nested in Repeat-2b |
| Repeat-5c | 188 | 454920 | 454733 | minus |  |
| Repeat-6a | 161 | 372429 | 372589 | plus |  |
| Repeat-6b | 161 | 403591 | 403751 | plus |  |
| Repeat-7a | 145 | 91676 | 91820 | plus |  |
| Repeat-7b | 145 | 295655 | 295511 | minus | nested in Repeat-2a |
| Repeat-7c | 145 | 361347 | 361203 | minus | nested in Repeat-2b |
| Repeat-8a | 113 | 12922 | 13034 | plus |  |
| Repeat-8b | 113 | 176472 | 176360 | minus |  |
| Repeat-9a | 112 | 244520 | 244631 | plus |  |
| Repeat-9b | 112 | 411570 | 411459 | minus |  |
| Repeat-10a | 90 | 244586 | 244675 | plus |  |
| Repeat-10b | 90 | 438042 | 437953 | minus |  |
| Repeat-11a | 88 | 93705 | 93792 | plus |  |
| Repeat-11b | 88 | 160105 | 160192 | plus |  |
| Repeat-12a | 82 | 32557 | 32638 | plus |  |
| Repeat-12b | 82 | 253447 | 253528 | plus |  |
| Repeat-13a | 81 | 179082 | 179162 | plus |  |
| Repeat-13b | 81 | 324742 | 324822 | plus |  |
| Repeat-14a | 72 | 360700 | 360629 | minus |  |
| Repeat-14b | 72 | 160670 | 160741 | plus |  |
| Repeat-15a | 72 | 160597 | 160668 | plus |  |
| Repeat-15b | 72 | 360773 | 360702 | minus |  |
| Repeat-16a | 69 | 296212 | 296280 | plus | nested in Repeat-2a |
| Repeat-16b | 69 | 361904 | 361972 | plus | nested in Repeat-2b |
| Repeat-16c | 69 | 404810 | 404878 | plus |  |
| Repeat-17a | 68 | 91607 | 91674 | plus |  |
| Repeat-17b | 68 | 295724 | 295657 | minus | nested in Repeat-2a |
| Repeat-17c | 68 | 361416 | 361349 | minus | nested in Repeat-2b |
| Repeat-18a | 66 | 177892 | 177957 | plus |  |
| Repeat-18b | 66 | 177925 | 177990 | plus |  |
| Repeat-19a | 62 | 283013 | 283074 | plus |  |
| Repeat-19b | 62 | 441731 | 441792 | plus |  |
| Repeat-20a | 59 | 238113 | 238171 | plus |  |
| Repeat-20b | 59 | 266741 | 266799 | plus |  |
| Repeat-21a | 56 | 295780 | 295835 | plus | nested in Repeat-2a |
| Repeat-21b | 56 | 361472 | 361527 | plus | nested in Repeat-2b |
| Repeat-21c | 56 | 404378 | 404433 | plus |  |
| Repeat-22a | 55 | 330091 | 330145 | plus |  |
| Repeat-22b | 55 | 438448 | 438502 | plus |  |
| Repeat-23a | 55 | 233857 | 233911 | plus |  |
| Repeat-23b | 55 | 261617 | 261563 | minus |  |
| Repeat-24a | 55 | 162932 | 162986 | plus |  |
| Repeat-24b | 55 | 377149 | 377203 | plus |  |
| Repeat-25a | 54 | 77107 | 77160 | plus |  |
| Repeat-25b | 54 | 177729 | 177782 | plus |  |
| Repeat-26a | 53 | 66974 | 67026 | plus | nested in Repeat-4a |
| Repeat-26b | 53 | 171619 | 171671 | plus |  |
| Repeat-26c | 53 | 373020 | 372968 | minus | nested in Repeat-4b |
| Repeat-27a | 53 | 107804 | 107752 | minus |  |
| Repeat-27b | 53 | 359189 | 359137 | minus |  |
| Repeat-27c | 53 | 3044 | 3096 | plus |  |
| Repeat-28a | 50 | 282003 | 282052 | plus |  |
| Repeat-28b | 50 | 343892 | 343941 | plus |  |

Supplementary Table 2: Rates of the rearrangement mediated by repeat pairs. Rates were estimated by ONT read mapping. IR and DR stand for Inverted repeat and direct repeat, respectively. Numbers in parenthesis in the rate columns indicate the number of supporting reads.

| **repeat pair** | **repeat length** | **%reference** | **%alternative** |
| --- | --- | --- | --- |
| Repeat-1a_b (IR) | 8989 | 48.28 (42) | 51.72 (45) |
| Repeat-2a_b (DR) | 1467 | 29.09 (32) | 70.91 (78) |
| Repeat-3a_b (IR) | 564 | 98.58 (139) | 1.42 (2) |
| Repeat-4a_b (IR) | 317 | 99.01 (200) | 0.99 (2) |
| Repeat-5a_c (IR) | 188 | 97.96 (96) | 2.04 (2) |
| Repeat-5b_c (IR) | 188 | 1.00 (107) | 0.00 (0) |
| Repeat-6a_b (DR) | 161 | 99.39 (164) | 0.61 (1) |

Supplementary Table 3: The numbers of mitochondrial reads in the HiFi and ONT datasets. The numbers of both fully mapped and non-fully mapped reads are shown.

|  | Fully and linearly mapped to MC1/MC2^2^ | Mappable to MC1/MC2^2^, but not fully | Total numbers of mitochondrial reads |
| --- | --- | --- | --- |
| HiFi | 27508 | 1531 | 29039 |
| ONT | 6583 | 343 | 6926 |
